# Mammographic features at primary breast cancer diagnosis in relation to recurrence-free survival

**DOI:** 10.1016/j.breast.2024.103736

**Published:** 2024-04-18

**Authors:** Kristina Lång, Li Sturesdotter, Ylva Bengtsson, Anna-Maria Larsson, Hanna Sartor

**Affiliations:** a Department of Translational Medicine, Diagnostic Radiology, Lund University, Lund, Sweden; b Department of Medical Imaging and Physiology, Skåne University Hospital, Lund/Malmö, Sweden; c Department of Clinical Sciences Lund, Division of Oncology, Lund University, Lund, Sweden; d Unilabs Breast Unit, Skåne University Hospital, Malmö, Sweden

**Keywords:** Breast cancer, Epidemiology, Mammography, Breast density, Recurrence, Prognosis

## Abstract

**Purpose:**

The number of women living with breast cancer (BC) is increasing, and the efficacy of surveillance programs after BC treatment is essential. Identification of links between mammographic features and recurrence could help design follow up strategies, which may lead to earlier detection of recurrence. The aim of this study was to analyze associations between mammographic features at diagnosis and their potential association with recurrence-free survival (RFS).

**Methods:**

Women with invasive BC in the prospective Malmö Diet and Cancer Study (n = 1116, 1991–2014) were assessed for locoregional and distant recurrences, with a median follow-up of 10.15 years. Of these, 34 women were excluded due to metastatic disease at diagnosis or missing recurrence data. Mammographic features (breast density [BI-RADS and clinical routine], tumor appearance, mode of detection) and tumor characteristics (tumor size, axillary lymph node involvement, histological grade) at diagnosis were registered. Associations were analyzed using Cox regression, yielding hazard ratios (HR) with 95 % confidence intervals (CI).

**Results:**

Of the 1082 women, 265 (24.4 %) had recurrent disease. There was an association between high mammographic breast density at diagnosis and impaired RFS (adjusted HR 1.32 (0.98–1.79). In analyses limited to screen-detected BC, this association was stronger (adjusted HR 2.12 (1.35–3.32). There was no association between mammographic tumor appearance and recurrence.

**Conclusion:**

RFS was impaired in women with high breast density compared to those with low density, especially among women with screen-detected BC. This study may lead to insights on mammographic features preceding BC recurrence, which could be used to tailor follow up strategies.

## Highlights

• Recurrence-free survival studied in relation to breast density and tumor appearances.
• Large study within a prospective population-based cohort with long-term follow-up.
• Evidence of impaired recurrence-free survival for women with dense breasts.
• No impaired recurrence-free survival due to mammographic tumor appearances.

## 1 Introduction

The prevalence of breast cancer (BC) is increasing due to an increase in incidence combined with an improved 5-year survival rate exceeding 90 % [1,2]. To detect a recurrence or a new contralateral second primary BC [3], an effective surveillance program is essential after BC treatment [4,5]. The general goal of the surveillance is to detect local recurrence or contralateral cancer, to treat therapy-related complications, and to assure patient wellbeing [5]. In the asymptomatic patient with early-stage BC no further imaging (e.g., computed tomography) is recommended to scan for distant recurrences, unless symptoms suggest otherwise [6]. However, patients with more advanced BC at diagnosis or who may be subject to targeted oncological therapies may be subject to additional imaging and follow-up to find distant recurrences at an early stage [7]. There are key tumor factors known to be associated with recurrence such as metastatic lymph-node involvement [6], high histological grade [8], and triple-negative cancers, the latter with the risk of early recurrence [6,9]. In addition, recurrence patterns vary between molecular subtypes, with Luminal A having the lowest rate of any type of recurrence [9]. Current guidelines recommend all patients annual mammography after BC treatment [10], and, thus, do not take individual risk of recurrence into account, which is a shortcoming. In terms of mammographic features, associations have been shown between higher breast density and risk of a locoregional recurrence, but not with distant recurrence [[11], [12], [13], [14]]. In addition, a previous large study identified both heterogeneously dense breasts and interval primary breast cancer as a predictor of an interval second breast cancer [15]. This raises questions on whether those with an increased risk of recurrence should undergo supplemental imaging. Further, to the best of our knowledge, no previous studies have investigated potential relations between mammographic tumor appearance and recurrence-free survival (RFS), and importantly, no previous studies have investigated several mammographic features combined. From a radiological point of view, mammographic breast density, mammographic tumor appearance, and the mode of detection (such as screening or clinical detection) have various degrees of prognostic information in the context of primary BC [[16], [17], [18]]. Identifying potential links between several mammographic features and RFS could help with the design of imaging strategies for patients at higher risk of recurrence. The aim of this study was to investigate three mammographic features at primary BC diagnosis and their potential association with RFS in the large prospective Malmö Diet and Cancer Study (MDCS).

## 2 Methods

### 2.1 Study population

The MDCS [[19], [20], [21]] included inhabitants of Malmö from the period of 1991–1996, of which 17,035 were women. The MDCS cohort includes information on vital status, causes of death, and cancer diagnoses, and is regularly updated according to the Swedish Cancer Registry and the Swedish Cause of Death Registry. All women in the cohort with BC diagnosed from 1991 until the end of 2014 were identified. Women with previous BC (n = 576), bilateral BC (n = 21), or non-invasive cancer (n = 105) were excluded. Further, 16 patients were excluded due to metastatic disease at initial BC diagnosis, and 18 patients were excluded due to missing information on BC recurrence. A total of 1082 women remained eligible for inclusion after the application of exclusion criteria. The data examined comprised baseline information (including use of hormone replacement therapy (HRT) and body mass index (BMI)), and factors related to BC diagnosis: mammographic information, pathological information, and data from medical records. Data on recurrent BC (endpoint data) were collected from medical records, pathology reports, and radiology reports. Recurrence was defined as locoregional or distant recurrence (17 % locoregional, 16 % locoregional and distant, 67 % distant only). Second primary (i.e., contralateral BC recurrence) was reported as distant recurrence. Patients were not followed up until a fixed date, instead, we used the date of the last medical record check in 2019 or early 2020 as the last date of follow-up for recurrent disease (median follow-up time 10.15 years). Patients were censored if they died or emigrated. The study was carried out in accordance with the Declaration of Helsinki and was approved by the Ethics Review Board in Lund, Sweden (Official Records Nos. 652/2005, 166/2007 and 2014/830) and by the Swedish Ethical Review Authority (2022-04473-02). The study includes a subset of the women in the MDCS cohort, and their informed consent was obtained at the baseline.

### 2.2 Clinical information

Detailed information on the mode of detection, image parameters, and tumor variables in the MDCS, BC cohort have been described previously in recent publication [16]. The mode of detection (screening or clinical detection), breast density, and tumor appearance were retrospectively collected from the diagnostic mammography report. Interval cancers were included in the group of clinically detected cancers. In our radiological department, density is routinely classified qualitatively using three categories: fat-involuted, moderately dense, and dense breast parenchyma. These categories correspond to The Breast Imaging Reporting and Data System (BI-RADS) 4th edition [22]: “fat involuted” corresponds to BI-RADS 1 (almost entirely fatty), “moderately dense” corresponds to BI-RADS 2 and 3 (scattered fibroglandular density and heterogeneously dense), and “dense” corresponds to BI-RADS 4 (extremely dense). For the latter part of the population (n = 376) diagnosed in 2008–2014, an additional density assessment according to BI-RADS 5th edition was made retrospectively (a = almost entirely fatty, b = scattered areas of fibroglandular density, c = heterogeneously dense, and d = extremely dense). The mammographic tumor appearances were divided into five groups according to an adjusted categorization by Luck et al. [23]: distinct mass, ill-defined mass, spiculated mass, calcification, and tissue abnormality (a combination of the two groups with architectural distortion and asymmetric tissue component). Histological grade was re-evaluated by a senior pathologist for the period of 1991–2004 and collected from medical records for the period of 2005 and onwards. Tumor size and axillary lymph node involvement (ALNI) were collected from medical records. Estrogen receptor (ER) status was collected from medical records or tissue microarrays (TMA) constructed for the MDCS cohort and dichotomized into positive (>10 % nuclear staining intensity) and negative (≤10 % nuclear staining intensity) [24].

### 2.3 Statistical analyses

Descriptive statistics were used to analyze the study population's characteristics in relation to recurrence. Kaplan-Meier estimate, and Cox's proportional hazard regression were used to study the impact of breast density and tumor appearance on RFS. The proportional hazards assumption was confirmed using a log-minus-log plot. Cox regression yielded hazard ratios (HRs) and 95 % confidence intervals (CIs). Analyses were performed for the whole population and then separately for screen- and clinically detected BC. Cox regression regarding breast density were performed with the two least dense categories combined (fat involuted + moderately dense parenchyma) compared to the densest category (dense breast parenchyma). Analyses were also performed separately for women with density assessment according to BI-RADS 5th edition (n = 376). These analyses were done using combinations of categories a & b and c & d. Analyses on density were adjusted for BMI, HRT, age at diagnosis, tumor size, ALNI, histological grade, and ER status. Analyses of tumor appearance (categorical, five groups) were adjusted for breast density, age at diagnosis, tumor size, ALNI, histological grade, and ER status. For adjustments, age at diagnosis and BMI were used as continuous variables, tumor size was dichotomized (≤20 mm or > 20 mm), ALNI was binarized (present or not), histological grade was divided into three ordinal categories, ER was binarized (negative or positive), HRT was binarized, and breast density was divided into three ordinal categories based on clinical practice. P values should be interpreted as evidence against the null hypothesis of no association without reference to a threshold for significance [25,26]. Missing values in the variables made up only a small portion of all values, and therefore Cox regression models were computed using complete cases. All calculations were performed with R version 4.2.2.

## 3 Results

**Fig. 1:**
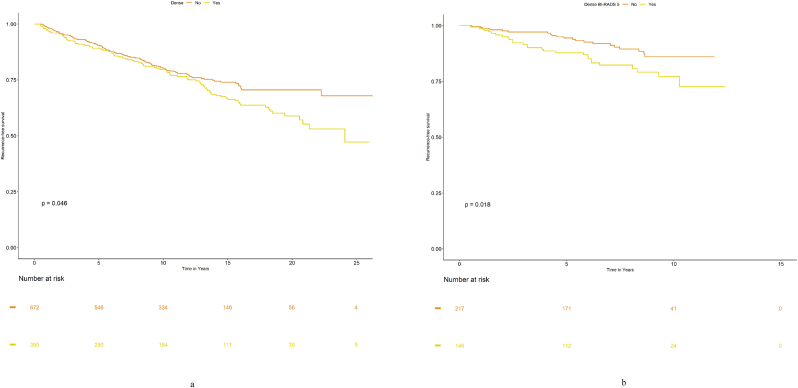
Fig. 1a: Kaplan-Meier graph shows RFS in relation to breast density (no/yes i.e., low/high breast density) Fig. 1b: Kaplan-Meier graph shows RFS in relation to BI-RADS breast density (no/yes i.e., category a and b/category c and d).

Table 1 presents the population characteristics in relation to BC recurrence. The median follow-up time was 10.15 years (range 0.04–27.87 years). A total of 265 women were diagnosed with a recurrence, and their median follow-up time was 5.85 years. For the remaining 817 women, the median follow-up time was 11.33 years. A spiculated mass was the most common tumor appearance (in terms of percentages) in women with and without recurrence, and a distinct mass were the second most common tumor appearance in both groups. Compared to women without recurrence, a greater proportion of women with recurrence had high breast density, were diagnosed clinically, and had more severe tumor characteristics in terms of large size, histological grade III, and ALNI. *Breast density* Two Kaplan-Meier graphs (Fig. 1a and b) show RFS in relation to breast density (low/high). The two density groups show a difference in the association with RFS for both the analysis with clinically measured density, available for the entire study period (p = 0.046, Fig. 1a), and the analysis with density measured according to BI-RADS, available for the latter half of the study period (p = 0.018, Fig. 1b). In the full-population Cox regression (Table 2), there was an association between high breast density and impaired RFS in the unadjusted analysis (HR 1.29 (95 % CI 1.00–1.67)), as well as in the adjusted analyses (HR_adj_ 1.32 (95 % CI 0.98–1.79)). In further analyses stratified by the mode of detection, in screen-detected cancers, there was a strong association between high breast density and impaired RFS (HR_adj_ 2.12 (95 % CI 1.35–3.32)). In clinically detected cancers, there was a weak inverse association between high density and impaired RFS (HR_adj_ 0.87 (95 % CI 0.56–1.34). All density analyses were adjusted for age at diagnosis, BMI, HRT, tumor size, ALNI, histological grade, and ER status.

**Table 1:** Patient characteristics and recurrence.

n	No recurrence	Recurrence	Total
	817	265	1082
Follow up time, years (median [range])	11.33 [0.04, 27.87]	5.85 [0.20, 24.09]	10.15 [0.04, 27.87]
Missing	0	0	0
Age at diagnosis, (median [range])	67 [46,91]	64 [45, 83]	66 [45, 91]
Missing	0	0	0
BMI, kg/m^2^ (median [range])	24.84 [16.23, 42.06]	25.16 [17.06, 41.41]	24.92 [16.23, 42.06]
Missing	0	0	0
HRT			
No	610 (74.9)	185 (69.8)	795 (73.7)
Yes	204 (25.1)	80 (30.2)	284 (26.3)
Missing	3	0	3
Estrogen receptor			
≤10 %	73 (10.0)	33 (14.7)	106 (11.1)
>10 %	656 (90.0)	191 (85.3)	847 (88.9)
Missing	88	41	129
Histological grade			
I	219 (28.8)	49 (20.1)	268 (26.7)
II	373 (49.1)	101 (41.4)	474 (47.2)
III	168 (22.1)	94 (38.5)	262 (26.1)
Missing	57	21	78
Mode of detection			
Screen-detection	420 (51.8)	125 (47.5)	545 (50.7)
Clinical detection	391 (48.2)	138 (52.5)	529 (49.3)
Missing	6	2	8
Axillary lymph node involvement			
No	535 (72.9)	136 (54.6)	671 (68.3)
Yes	199 (27.1)	113 (45.4)	312 (31.7)
Missing	83	16	99
Tumor size			
≤20 mm	583 (74.6)	159 (61.2)	742 (71.3)
>20 mm	198 (25.4)	101 (38.8)	299 (28.7)
Missing	36	5	41
Mammographic appearance			
Distinct mass	202 (26.8)	55 (23.7)	257 (26.1)
Ill-defined mass	151 (20.0)	47 (20.3)	198 (20.1)
Spiculated mass	321 (42.6)	89 (38.4)	410 (41.6)
Calcifications	52 (6.9)	31 (13.4)	83 (8.4)
Tissue abnormality	28 (3.7)	10 (4.3)	38 (3.9)
Missing	63	33	96
Breast density			
Fat involuted	149 (19.2)	31 (12.7)	180 (17.6)
Moderately dense	382 (49.1)	110 (45.1)	492 (48.1)
Dense	247 (31.7)	103 (42.2)	350 (34.2)
Missing	39	21	60
BI-RADS 5			
Almost entirely fatty	77 (24.4)	7 (14.6)	84 (23.1)
Scattered areas of fibroglandular density	119 (37.8)	14 (29.2)	133 (36.6)
Heterogeneously dense	98 (31.1)	19 (39.6)	117 (32.2)
Extremely dense	21 (6.7)	8 (16.7)	29 (8.0)
Not evaluated	502	217	719

**Table 2:** Breast density in relation to recurrence.

Variable	HR (95 % CI)	p-value	HR^a^ (95 % CI)	p-value
Breast density		0.047		0.071
Fat involuted/Moderately dense	1.0 (Reference)		1.0 (Reference)	
Dense	1.29 (1.00–1.67)		1.32 (0.98–1.79)	
Screen-detection				
Breast density		<0.001		0.001
Fat involuted/Moderately dense	1.0 (Reference)		1.0 (Reference)	
Dense	1.99 (1.4–2.85)		2.12 (1.35–3.32)	
Clinical detection				
Breast density		0.257		0.521
Fat involuted/Moderately dense	1.0 (Reference)		1.0 (Reference)	
Dense	0.81 (0.56–1.17)		0.87 (0.56–1.34)	

a Adjusted for BMI, HRT, age at diagnosis, tumour size, lymph node involvement, histological grade, and oestrogen receptor.

Table 3 and Supplementary Tab 1 (all covariates) shows the results of multivariable analyses for the women who were also assessed with density measurements according to BI-RADS 5th edition. These analyses showed strong associations between high density and impaired RFS, with a similar distribution and magnitude to those in the full-population results in Table 2. Specifically, the HR and HR_adj_ of recurrence for the group with high breast density were 1.97 (95 % CI 1.11–3.48) and 1.73 (95 % CI 0.92–3.25), respectively, compared to the low breast density group. In analyses that were limited to screen-detected cancers, the HR_adj_ of recurrence for the high-breast-density group was 3.23 (95 % CI 1.17–8.93). For women with clinically detected cancer, no association was found between density and RFS. *Tumor appearance*.

**Table 3:** BI-RADS 5th density measurement in relation to recurrence.

Variable	HR (95 % CI)	p-value	HR^a^ (95 % CI)	p-value
BI-RADS 5		0.020		0.089
Almost entirely fatty/Scattered areas of fibroglandular density	1.0 (Reference)		1.0 (Reference)	
Heterogeneously dense/Extremely dense	1.97 (1.11–3.48)		1.73 (0.92–3.25)	
Screen-detection				
BI-RADS 5		0.007		0.024
Almost entirely fatty/Scattered areas of fibroglandular density	1.0 (Reference)		1.0 (Reference)	
Heterogeneously dense/Extremely dense	3.72 (1.44–9.6)		3.23 (1.17–8.93)	
Clinical detection				
BI-RADS 5		0.695		0.541
Almost entirely fatty/Scattered areas of fibroglandular density	1.0 (Reference)		1.0 (Reference)	
Heterogeneously dense/Extremely dense	1.15 (0.56–2.37)		1.31 (0.55–3.09)	

a Adjusted for BMI, HRT, age at diagnosis, tumour size, axillary lymph node involvement, histological grade, and oestrogen receptor.

**Fig. 2:**
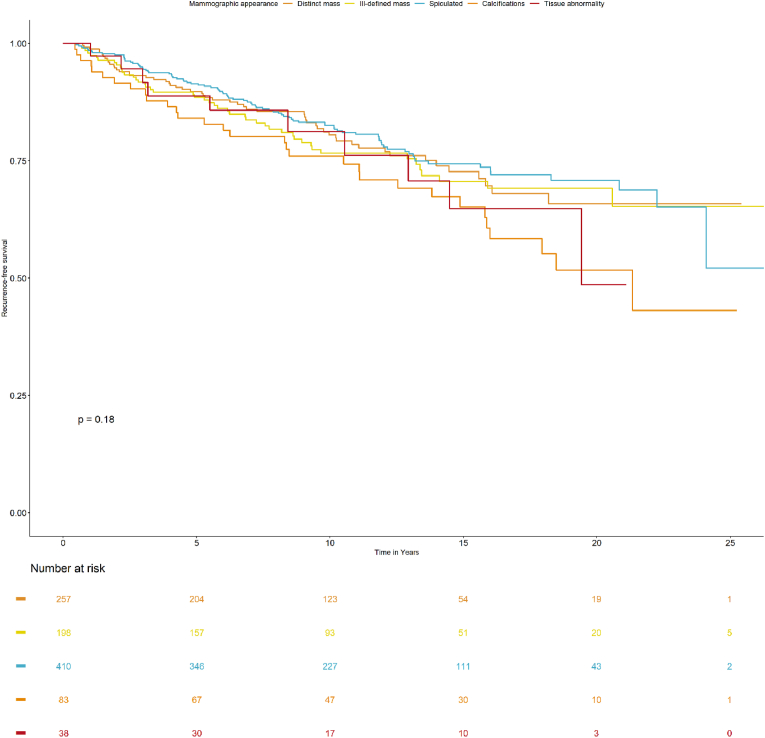
Kaplan-Meier graph shows RFS in relation mammographic tumor appearance.

Fig. 2 shows a Kaplan-Meier graph for the full study population in relation to tumor appearances. The different groups of tumor appearance showed no evidence of an association with RFS (p = 0.18). In a subsequent Cox regression (Table 4), HR and HR_adj_ showed no association between tumor appearance and impaired RFS. In the stratified unadjusted analysis on screen-detected cancers, there was an inverse relation between spiculated tumor masses and impaired RFS (overall p = 0.033; HR 0.66 (95 % CI 0.41–1.05)). However, after adjustments, this association was weak (overall p = 0.415; HR_adj_ 0.76 (95 % CI 0.44–1.32)). No association between tumor appearance and RFS was detected among clinically detected cancers. All analyses regarding tumor appearance were adjusted for breast density, age at diagnosis, tumor size, ALNI, histological grade, and ER status.

**Table 4:** Mammographic tumor appearance in relation to recurrence.

Variable	HR (95 % CI)	p-value	HR^a^ (95 % CI)	p-value
Mammographic appearance		0.221		0.589
Distinct mass	1.0 (Reference)		1.0 (Reference)	
Ill-defined mass	1.06 (0.72–1.57)		1.05 (0.67–1.64)	
Spiculated	0.92 (0.66–1.29)		1.08 (0.73–1.59)	
Calcifications	1.52 (0.98–2.37)		1.58 (0.91–2.75)	
Tissue abnormality	1.24 (0.63–2.42)		1.28 (0.59–2.78)	
Screen-detection				
Mammographic appearance		0.033		0.415
Distinct mass	1.0 (Reference)		1.0 (Reference)	
Ill-defined mass	0.61 (0.33–1.14)		0.74 (0.36–1.56)	
Spiculated	0.66 (0.41–1.05)		0.76 (0.44–1.32)	
Calcifications	1.39 (0.80–2.39)		1.38 (0.69–2.76)	
Tissue abnormality	0.70 (0.21–2.32)		0.59 (0.14–2.62)	
Clinical detection				
Mammographic appearance		0.270		0.374
Distinct mass	1.0 (Reference)		1.0 (Reference)	
Ill-defined mass	1.65 (0.98–2.77)		1.4 (0.76–2.56)	
Spiculated	1.48 (0.91–2.4)		1.72 (0.97–3.05)	
Calcifications	1.82 (0.79–4.21)		1.32 (0.44–3.91)	
Tissue abnormality	1.84 (0.80–4.25)		2.16 (0.81–5.72)	

a Adjusted for breast density, age at diagnosis, tumour size, axillary lymph node involvement, histological grade, and oestrogen receptor.

## 4 Discussion

In this retrospective study on 1082 Swedish women with invasive BC from the prospective Malmö Diet and Cancer study we found an association between higher breast density and impaired RFS, especially for BC detected by screening. There was no evidence of an association between mammographic tumor appearance and RFS, neither in the full population, nor in the analyses stratified by mode of detection. Previous studies have found a positive associations between higher density and locoregional recurrence [12,13] and later development of contralateral cancer [27], but not with distant recurrence [12,13]. Conversely, a lack of association between density and adverse outcomes (such as loco-regional recurrence, distant metastasis, or death caused by BC or any other cause) have also been reported [28,29]. Comparison between studies is challenging because of the heterogeneity of study populations and choice of density measurements. Furthermore, endpoint definitions also vary between studies which is a known complicating factor [3]. In this present study, our aim was to analyze density measurements in relation to all types of recurrence to provide a broader understanding of a possible association. The link between density and RFS was particularly evident in BC detected by screening. The reason for this is not clear. If an association exists also in the group of women with clinically detected cancer, it might be difficult to detect due to the heterogeneity in this subgroup (including age outside the ranges for screening programs, and symptomatic cancers, including interval cancers). In addition, it is known from oncological BC research that prognostic models perform suboptimal in high-risk populations [30]. One may hypothesize that is more difficult to detect the effect of a single prognostic variable, such as breast density, in a more complex high risk cancer, such as a clinically detected cancer known for a worse prognosis [31]. Interestingly, in a recent study from our research group on density and BC survival [16], women with BC detected by screening had the strongest evidence for a link between high density and impaired survival, although the result was not statistically significant. A biological reason for high breast density being especially harmful in women with tumors detected by screening is difficult to explain, and the results need to be interpreted with care. The association of high density and recurrence could in part be explained by the masking effect [32] of dense tissue leading to a later detection, higher stage at diagnosis, and thereby a worse prognosis. However, by adjusting for tumor size, axillary lymph node involvement, and histological grade, we believe we have taken the masking into account in analyses, but some residual effect cannot be excluded. To the best of our knowledge, there have been no previous studies on the association between different types of mammographic tumor appearance and RFS, which makes this study an important contribution from a radiological point of view. Two previous studies have investigated a specific branching type of microcalcifications in relation to disease-free survival and have concluded that mammographic casting-type calcification is an independent poor prognostic factor in invasive BC. However, breast density or other tumor appearances has not been considered in these two reports [33,34]. Even if not the scope of this present study, two small previous studies have compared the tumor appearance between primary and recurrent BC and found some evidence of resemblance, highlighting the importance of reviewing pretreatment mammograms during follow-up of patients [35,36]. In the present study, we found no evidence of an association between mammographic tumor appearances and RFS, neither in the full population nor in analyses stratified by mode of detection. This is in line with the lack of association previously observed between mammographic tumor appearance and BC survival [16]. It might be that in certain subgroups of BC with a distinct tumor appearance (such as the aggressive triple negative BC [9,37]), there could be a link between tumor appearance and early, rather than late, recurrence [9]. Larger populations are needed to perform such sub-analyses. During the past decade, high breast density in BC screening and diagnosis has been thoroughly studied, and some consensus has been reached on how to manage density in population based screening [38]. However, there is insufficient understanding of the role of density and other imaging factors, such as the mode of detection and mammographic tumor appearance, in follow-up care including surveillance programs with breast imaging. In terms of personalized BC follow-up, the INFLUENCE prediction model has been developed and validated by a Dutch research team [39,40]. The latest version of the model shows great ability to identify patients with a low or high risk of recurrence (i.e., locoregional recurrence, distant recurrence, second primary BC), based on grade, size, multifocality, ALNI, and potential treatment with radio-, chemo- or hormone therapy. The addition of image factors to such a model would be particularly interesting. The present study could add knowledge about which factors should be included in such a model, for example breast density. This study has several strengths. Firstly, the population was large and included both clinical and radiological information. Secondly, the follow-up time was long and endpoint data was retrieved from high-quality Swedish national registries [[41], [42], [43]]. However, there are also a few methodological considerations. Women in the MDCS are generally healthier and have a slightly higher educational level than the average female population in Sweden, which may affect the representativeness of the results [20]. Nevertheless, this should not affect internal comparisons within the MDCS cohort. Mode of treatment (surgical and oncological) has varied over the long study period but was not included as an adjustment factor in this study, this is also in line with previous MDCS studies. However, by adjusting for selected prognostic tumor characteristics, which are the base for treatment selection independent of time period, we propose our statistical models valid over this long-time span. However, some residual confounding cannot be excluded. With a larger population size, it would have been interesting to adjust for calendar time, but this was not possible in this present study. Qualitative assessment of mammograms regarding breast density and tumor appearance may be limited by inter-reader variability. But at the same time, this variation reflects everyday clinical practice in a department of breast radiology which means the results may be more generalizable.

In conclusion, high breast density at the time of primary BC diagnosis was associated with impaired RFS for women within the MDCS cohort. This was especially evident for women with screen-detected cancers. We found no association between mammographic tumor appearance and RFS. This study may lead to insights on risk factors related to tumor biology, mammographic patterns at diagnosis, and BC recurrence, which in turn could be used to tailor surveillance.

## Data statement

The dataset from the Malmö Diet and Cancer Study supporting the conclusions of this article was used under a license and is not available as an open source.

## Ethics approval

This study was carried out in accordance with the declaration of Helsinki. It was approved by the Ethics Review Board in Lund, Sweden (Official Records Nos. 652/2005, 166/2007 and 2014/830) and by the Swedish Ethical Review Authority (2022-04473-02). It includes a subset of the women in the MDCS cohort, and their informed consent was obtained at baseline.

## Funding

This work was supported by Lions Research Fund, 10.13039/100016409Gyllenstiernska Krapperup Foundation, and governmental funding within the NHS.

## CRediT authorship contribution statement

**Kristina Lång:** Conceptualization, Data curation, Methodology, Writing – original draft, Writing – review & editing. **Li Sturesdotter:** Data curation, Investigation, Methodology, Writing – review & editing. **Ylva Bengtsson:** Data curation, Investigation, Methodology, Writing – review & editing. **Anna-Maria Larsson:** Conceptualization, Methodology, Writing – review & editing. **Hanna Sartor:** Conceptualization, Data curation, Formal analysis, Funding acquisition, Investigation, Methodology, Project administration, Resources, Software, Supervision, Validation, Visualization, Writing – original draft, Writing – review & editing.

## References

[1] Arnold M., Morgan E., Rumgay H., Mafra A., Singh D., Laversanne M. (2022). Current and future burden of breast cancer: global statistics for 2020 and 2040. Breast.

[2] Miller K.D., Nogueira L., Devasia T., Mariotto A.B., Yabroff K.R., Jemal A. (2022). Cancer treatment and survivorship statistics, 2022. CA A Cancer J Clin.

[3] Moossdorff M., van Roozendaal L.M., Strobbe L.J., Aebi S., Cameron D.A., Dixon J.M. (2014). Maastricht Delphi consensus on event definitions for classification of recurrence in breast cancer research. J Natl Cancer Inst.

[4] Houssami N., Ciatto S. (2010). Mammographic surveillance in women with a personal history of breast cancer: how accurate? How effective?. Breast.

[5] Høeg B.L., Bidstrup P.E., Karlsen R.V., Friberg A.S., Albieri V., Dalton S.O. (2019). Follow-up strategies following completion of primary cancer treatment in adult cancer survivors. Cochrane Database Syst Rev.

[6] Senkus E., Kyriakides S., Ohno S., Penault-Llorca F., Poortmans P., Rutgers E. (2015). Primary breast cancer: ESMO Clinical Practice Guidelines for diagnosis, treatment and follow-up. Ann Oncol : official journal of the European Society for Medical Oncology / ESMO.

[7] Lother D., Robert M., Elwood E., Smith S., Tunariu N., Johnston S.R.D. (2023). Imaging in metastatic breast cancer, CT, PET/CT, MRI, WB-DWI, CCA: review and new perspectives. Cancer Imag.

[8] Rakha E.A., Reis-Filho J.S., Baehner F., Dabbs D.J., Decker T., Eusebi V. (2010). Breast cancer prognostic classification in the molecular era: the role of histological grade. Breast Cancer Res.

[9] Park S., Koo J.S., Kim M.S., Park H.S., Lee J.S., Lee J.S. (2012). Characteristics and outcomes according to molecular subtypes of breast cancer as classified by a panel of four biomarkers using immunohistochemistry. Breast.

[10] Lam D.L., Houssami N., Lee J.M. (2017). Imaging surveillance after primary breast cancer treatment. AJR Am J Roentgenol.

[11] Huang Y.S., Chen J.L., Huang C.S., Kuo S.H., Jaw F.S., Tseng Y.H. (2016). High mammographic breast density predicts locoregional recurrence after modified radical mastectomy for invasive breast cancer: a case-control study. Breast Cancer Res.

[12] Park C.C., Rembert J., Chew K., Moore D., Kerlikowske K. (2009). High mammographic breast density is independent predictor of local but not distant recurrence after lumpectomy and radiotherapy for invasive breast cancer. Int J Radiat Oncol Biol Phys.

[13] Eriksson L., Czene K., Rosenberg L., Humphreys K., Hall P. (2013). Possible influence of mammographic density on local and locoregional recurrence of breast cancer. Breast Cancer Res.

[14] Cil T., Fishell E., Hanna W., Sun P., Rawlinson E., Narod S.A. (2009). Mammographic density and the risk of breast cancer recurrence after breast-conserving surgery. Cancer.

[15] Lee J.M., Buist D.S., Houssami N., Dowling E.C., Halpern E.F., Gazelle G.S. (2015). Five-year risk of interval-invasive second breast cancer. J Natl Cancer Inst.

[16] Sturesdotter L., Larsson A.-M., Zackrisson S., Sartor H. (2023). Investigating the prognostic value of mammographic breast density and mammographic tumor appearance in women with invasive breast cancer: the Malmö Diet and cancer study. Breast.

[17] Sturesdotter L., Sandsveden M., Johnson K., Larsson A.M., Zackrisson S., Sartor H. (2020). Mammographic tumour appearance is related to clinicopathological factors and surrogate molecular breast cancer subtype. Sci Rep.

[18] Shawky M.S., Huo C.W., Henderson M.A., Redfern A., Britt K., Thompson E.W. (2019). A review of the influence of mammographic density on breast cancer clinical and pathological phenotype. Breast Cancer Res Treat.

[19] Berglund G., Elmstahl S., Janzon L., Larsson S.A. (1993). The malmo Diet and cancer study. Design and feasibility. J Intern Med.

[20] Manjer J., Carlsson S., Elmstahl S., Gullberg B., Janzon L., Lindstrom M. (2001). The Malmo Diet and Cancer Study: representativity, cancer incidence and mortality in participants and non-participants. Eur J Cancer Prev.

[21] Manjer J., Elmstahl S., Janzon L., Berglund G. (2002). Invitation to a population-based cohort study: differences between subjects recruited using various strategies. Scand J Publ Health.

[22] D'Orsi C.J.S.E., Mendelson E.B., Morris E.A. (2013). Breast imaging reporting and data System.

[23] Luck A.A., Evans A.J., James J.J., Rakha E.A., Paish E.C., Green A.R. (2008). Breast carcinoma with basal phenotype: mammographic findings. AJR Am J Roentgenol.

[24] Bengtsson Y., Demircan K., Rosendahl A.H., Borgquist S., Sandsveden M., Manjer J. (2022). Zinc and breast cancer survival: a prospective cohort study of dietary intake and serum levels. Nutrients.

[25] Amrhein V., Greenland S., McShane B. (2019). Scientists rise up against statistical significance. Nature.

[26] Greenland S., Senn S.J., Rothman K.J., Carlin J.B., Poole C., Goodman S.N. (2016). Statistical tests, P values, confidence intervals, and power: a guide to misinterpretations. Eur J Epidemiol.

[27] Knight J.A., Blackmore K.M., Fan J., Malone K.E., John E.M., Lynch C.F. (2018). The association of mammographic density with risk of contralateral breast cancer and change in density with treatment in the WECARE study. Breast Cancer Res.

[28] Gierach G.L., Ichikawa L., Kerlikowske K., Brinton L.A., Farhat G.N., Vacek P.M. (2012). Relationship between mammographic density and breast cancer death in the Breast Cancer Surveillance Consortium. J Natl Cancer Inst.

[29] Strand F., Humphreys K., Holm J., Eriksson M., Törnberg S., Hall P. (2018). Long-term prognostic implications of risk factors associated with tumor size: a case study of women regularly attending screening. Breast Cancer Res.

[30] Phung M.T., Tin Tin S., Elwood J.M. (2019). Prognostic models for breast cancer: a systematic review. BMC Cancer.

[31] Domingo L., Blanch J., Servitja S., Corominas J.M., Murta-Nascimento C., Rueda A. (2013). Aggressiveness features and outcomes of true interval cancers comparison between screen-detected and symptom-detected cancers. Eur J Cancer Prev.

[32] Wengert G.J., Helbich T.H., Leithner D., Morris E.A., Baltzer P.A.T., Pinker K. (2019). Multimodality imaging of breast parenchymal density and correlation with risk assessment. Curr Breast Cancer Rep.

[33] Li Y., Cao J., Zhou Y., Mao F., Shen S., Sun Q. (2019). Mammographic casting-type calcification is an independent prognostic factor in invasive breast cancer. Sci Rep.

[34] Peacock C., Given-Wilson R.M., Duffy S.W. (2004). Mammographic casting-type calcification associated with small screen-detected invasive breast cancers: is this a reliable prognostic indicator?. Clin Radiol.

[35] Günhan-Bilgen I., Oktay A. (2007). Mammographic features of local recurrence after conservative surgery and radiation therapy: comparison with that of the primary tumor. Acta Radiol.

[36] Philpotts L.E., Lee C.H., Haffty B.G., Lange R.C., Tocino I. (1996). Mammographic findings of recurrent breast cancer after lumpectomy and radiation therapy: comparison with the primary tumor. Radiology.

[37] Gao B., Zhang H., Zhang S.D., Cheng X.Y., Zheng S.M., Sun Y.H. (2014). Mammographic and clinicopathological features of triple-negative breast cancer. Br J Radiol.

[38] Tagliafico A.S., Houssami N. (2023). Towards consensus on managing high mammographic density in population breast screening?. Breast.

[39] Witteveen A., Vliegen I.M., Sonke G.S., Klaase J.M., Mj I.J., Siesling S. (2015). Personalisation of breast cancer follow-up: a time-dependent prognostic nomogram for the estimation of annual risk of locoregional recurrence in early breast cancer patients. Breast Cancer Res Treat.

[40] Voelkel V., Draeger T., Groothuis-Oudshoorn C.G.M., de Munck L., Hueting T., Gerken M. (2019). Predicting the risk of locoregional recurrence after early breast cancer: an external validation of the Dutch INFLUENCE-nomogram with clinical cancer registry data from Germany. J Cancer Res Clin Oncol.

[41] Johansson L.A., Björkenstam C., Westerling R. (2009). Unexplained differences between hospital and mortality data indicated mistakes in death certification: an investigation of 1,094 deaths in Sweden during 1995. J Clin Epidemiol.

[42] Barlow L., Westergren K., Holmberg L., Talbäck M. (2009). The completeness of the Swedish Cancer Register: a sample survey for year 1998. Acta Oncol.

[43] Brooke H.L., Talbäck M., Hörnblad J., Johansson L.A., Ludvigsson J.F., Druid H. (2017). The Swedish cause of death register. Eur J Epidemiol.

