# Supplementary material for: Mammographic features at primary breast cancer diagnosis in relation to recurrence-free survival
Source: Breast. 2024 Apr 18;75:103736. doi: 10.1016/j.breast.2024.103736 (PMC11068602; doi:10.1016/j.breast.2024.103736)
Supplement: Multimedia component 1 [file mmc1.docx]

Supplementary table 1: BI-RADS density with covariates, in relation to recurrence

|  | All | | Screen-detection | | Clinical detection | |
| --- | --- | --- | --- | --- | --- | --- |
| Variable | HR (95% CI) | p-value | HR (95% CI) | p-value | HR (95% CI) | p-value |
| BI-RADS 5 |  | 0.089 |  | 0.024 |  | 0.541 |
| Almost entirely fatty/Scattered areas of fibroglandular density | 1.0 (Reference) |  | 1.0 (Reference) |  | 1.0 (Reference) |  |
| Heterogeneously dense/Extremely dense | 1.73 (0.92-3.25) |  | 3.23 (1.17-8.93) |  | 1.31 (0.55-3.09) |  |
| Age at diagnosis | 0.995 (0.952-1.040) | 0.832 | 0.931 (0.816-1.060) | 0.293 | 0.998 (0.947-1.050) | 0.934 |
| BMI, kg/m2 | 0.971 (0.890-1.060) | 0.510 | 0.938 (0.811-1.090) | 0.391 | 0.983 (0.881-1.100) | 0.757 |
| HRT |  | 0.081 |  | 0.105 |  | 0.294 |
| No | 1.0 (Reference) |  | 1.0 (Reference) |  | 1.0 (Reference) |  |
| Yes | 1.81 (0.93-3.52) |  | 2.32 (0.84-6.44) |  | 1.64 (0.65-4.12) |  |
| Tumor size |  | 0.006 |  | 0.254 |  | 0.025 |
| ≤20 mm | 1.0 (Reference) |  | 1.0 (Reference) |  | 1.0 (Reference) |  |
| >20 mm | 2.56 (1.32-4.98) |  | 2.1 (0.588-7.47) |  | 2.85 (1.14-7.13) |  |
| Axillary lymph node involvement |  | 0.039 |  | 0.672 |  | 0.054 |
| No | 1.0 (Reference) |  | 1.0 (Reference) |  | 1.0 (Reference) |  |
| Yes | 2.0 (1.04-3.85) |  | 1.31 (0.378-4.52) |  | 2.33 (0.99-5.53) |  |
| Histological grade |  | 0.558 |  | 0.783 |  | 0.705 |
| I | 1.0 (Reference) |  | 1.0 (Reference) |  | 1.0 (Reference) |  |
| II | 0.99 (0.43-2.26) |  | 0.82 (0.28-2.42) |  | 1.32 (0.34-5.09) |  |
| III | 1.49 (0.59-3.73) |  | 1.44 (0.27-7.62) |  | 1.72 (0.44-6.74) |  |
| Estrogen receptor |  |  |  |  |  |  |
| ≤10% | 1.0 (Reference) |  | 1.0 (Reference) |  | 1.0 (Reference) |  |
| >10% | 1.50 (0.48-4.69) | 0.486 | 1.99 (0.18-22.2) | 0.575 | 1.33 (0.35-4.99) | 0.674 |
